# Identification of environmental sounds and melodies in syndromes of anterior temporal lobe degeneration^☆^

**DOI:** 10.1016/j.jns.2015.03.007

**Published:** 2015-05-15

**Authors:** Hannah L. Golden, Laura E. Downey, Philip D. Fletcher, Colin J. Mahoney, Jonathan M. Schott, Catherine J. Mummery, Sebastian J. Crutch, Jason D. Warren

**Affiliations:** Dementia Research Centre, UCL Institute of Neurology, University College London, London, United Kingdom

**Keywords:** Semantic, Environmental sounds, Music, Semantic dementia, Frontotemporal dementia, Frontotemporal lobar degeneration

## Abstract

Recognition of nonverbal sounds in semantic dementia and other syndromes of anterior temporal lobe degeneration may determine clinical symptoms and help to define phenotypic profiles. However, nonverbal auditory semantic function has not been widely studied in these syndromes. Here we investigated semantic processing in two key nonverbal auditory domains – environmental sounds and melodies – in patients with semantic dementia (SD group; n = 9) and in patients with anterior temporal lobe atrophy presenting with behavioural decline (TL group; n = 7, including four cases with MAPT mutations) in relation to healthy older controls (n = 20). We assessed auditory semantic performance in each domain using novel, uniform within-modality neuropsychological procedures that determined sound identification based on semantic classification of sound pairs. Both the SD and TL groups showed comparable overall impairments of environmental sound and melody identification; individual patients generally showed superior identification of environmental sounds than melodies, however relative sparing of melody over environmental sound identification also occurred in both groups. Our findings suggest that nonverbal auditory semantic impairment is a common feature of neurodegenerative syndromes with anterior temporal lobe atrophy. However, the profile of auditory domain involvement varies substantially between individuals.

## Highlights

• Nonverbal auditory processing is an understudied area of semantic memory.
• We assessed novel auditory semantic tasks in patients with temporal lobe atrophy.
• Environmental sound and music processing were comparably impaired across the cohort.
• Individual patients may show relative sparing of melody processing.
• Nonverbal auditory deficits integrally accompany temporal lobe degeneration.

## 1 Introduction

Semantic dementia (SD) is a focal neurodegenerative syndrome characterised by insidiously progressive impairment of semantic memory due to selective, asymmetric antero-medial temporal lobe atrophy [1]. SD is a canonical syndrome of frontotemporal lobar degeneration (FTLD) yet its phenotypic boundaries remain unclear: while semantic processing of words and visual objects has been studied intensively, much less is known concerning other knowledge modalities in SD that are likely also to contribute to symptoms and disability [1–9]. Furthermore, although research consensus diagnostic criteria have been developed for stratifying the major syndromes of FTLD [10,11], in practice the SD syndrome often shows substantial overlap clinically and anatomically with other syndromes of FTLD, in particular behavioural variant frontotemporal dementia (bvFTD) [12,13]. While not the presenting feature, semantic deficits are often prominent in such cases; moreover this spectrum includes cases with selective (particularly nondominant) temporal lobe atrophy [12–15] and the important subgroup represented by MAPT mutations, characteristically accompanied by focal bilateral anterior temporal lobe atrophy [12,16,17]. Conversely, SD is commonly associated with early and prominent behavioural abnormalities that overlap closely with bvFTD [18].

Among the nonvisual sensory modalities of semantic knowledge, nonverbal sound is of particular interest as a potential probe of this SD-like phenotypic spectrum, on both clinical and neuroanatomical grounds. The nonverbal auditory domain encompasses both environmental sound sources and events (including highly biologically and socially salient signals) and music (which exemplifies a nonverbal, abstract and autonomous rule-based semantic system [19,20]). Studies of auditory agnosia associated with focal brain damage and functional imaging studies of nonverbal sound processing in the healthy brain have implicated distributed temporo-parietal networks that closely overlap those damaged in FTLD [21–29]. The available evidence further suggests that these auditory semantic domains are at least partly dissociable neuropsychologically and neuroanatomically [21,30,31]. In line with this neuropsychological and neuroanatomical evidence, impaired identification of environmental sounds has been documented as part of more generalised semantic impairment in SD [2,4,7]. Findings for music processing in SD and other syndromes of FTLD appear somewhat inconsistent [3,5,32–35]: several well documented case studies show relatively-spared identification of melodies in patients with SD despite widespread semantic deficits [3,5,35], though group studies have qualified this [6,34]. In addition, a substantial proportion of patients with SD and bvFTD develop abnormal craving for music (‘musicophilia’) and/or aversion to environmental sounds [36,37], suggesting that these nonverbal auditory domains may underpin a repertoire of clinically salient symptoms in these disorders. However, previous neuropsychological investigations have relied largely on cross-modal labelling (word or picture matching) or in the case of melodies, familiarity judgement, rather than assessing specific within-modality identification and comparing domains directly. Accordingly, the true status of nonverbal sound processing in SD and related syndromes has not been fully defined.

Here we assessed nonverbal auditory semantic processing across the domains of environmental sounds and music in a cohort of patients with SD. In parallel, we assessed a group of patients presenting with behavioural symptoms (i.e., a syndrome of bvFTD) who also had selective temporal lobe atrophy on MRI. Our objectives were to assess in detail clinically-relevant domains of nonverbal auditory semantic memory in SD; and to compare the auditory semantic profile in SD with another syndrome (bvFTD) associated with anterior temporal lobe degeneration. Identification of environmental sounds and melodies was compared using a novel, uniform procedure based on within-modality stimulus matching, thereby obviating the need for cross-modal (especially, verbal) labelling. Based on previous neuropsychological and clinical evidence [2–6,35], we hypothesised that patients with SD would show impairments in both nonverbal auditory semantic domains, albeit less severely and consistently for melodies; that relative sparing of melody knowledge might be a hallmark of the SD group; and that patients with a syndrome of SD would show more severe nonverbal auditory semantic deficits than other patients with selective temporal lobe atrophy.

## 2 Methods

### 2.1 Participants

Nine consecutive patients fulfilling consensus criteria for typical SD [10] and seven patients who fulfilled consensus criteria for a primary syndrome of probable bvFTD [11] with predominant temporal lobe atrophy on MRI (based on visual assessment by an experienced neuroradiologist blinded to clinical diagnosis) were recruited from a specialist cognitive disorders clinic (representative sections from each patient's brain MRI are shown in Figure S1 in Supplementary material on-line). The latter, bvFTD syndromic group is hereafter designated the ‘temporal lobe’ [TL] group to emphasise the criterion of relatively focal temporal lobe atrophy used in selecting these patients; it is this subgroup that overlaps most closely with the SD group. The TL group included four patients with a confirmed pathogenic MAPT mutation; this high proportion is in line with previous evidence that these mutation cases usually present with behavioural changes but frequently also exhibit prominent semantic deficits associated with focal antero-medial temporal lobe atrophy [12,16,17]. Twenty age-matched healthy individuals also participated in the study. No participant had a history of clinical hearing loss. All participants underwent comprehensive assessment of musical background and general neuropsychological functions; participant group characteristics including background behavioural data are summarised in Table S1 in Supplementary Material on-line. None of the participants was a professional musician; on the basis of a previously described semi-structured caregiver questionnaire [36], one patient in the SD group and two in the TL group exhibited musicophilia, defined as abnormally increased interest in music compared with premorbid levels sufficient to interfere with daily life functioning.

### 2.2 Experimental behavioural tests

**Fig. 1:**
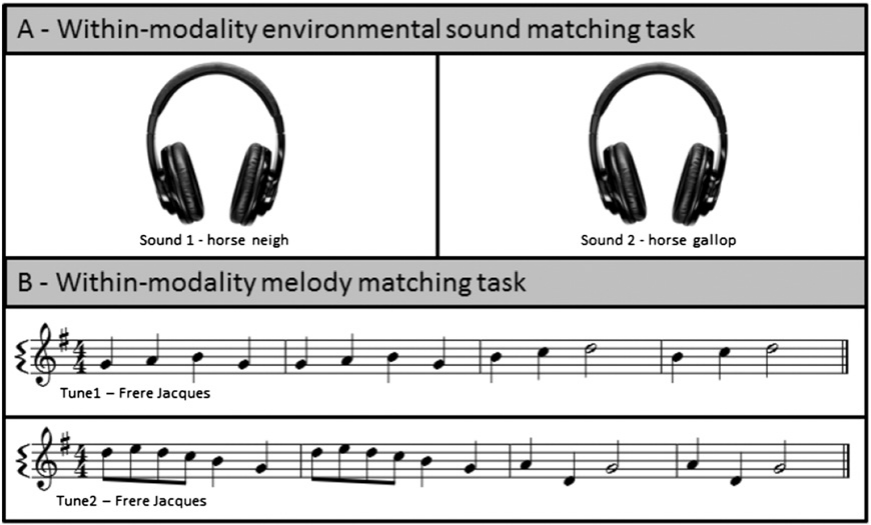
Examples of environmental sounds (A) and notated tune excerpts (B) used in the identification by within-modality matching tasks (here, examples for the ‘same’ source sound and melody conditions are shown).

We adapted a previously described paradigm [4,5] to create tests of environmental sound and melody identification within the auditory modality. These tests were based on presentation of pairs of sound stimuli derived either from the same environmental sound source or tune (‘same’ condition) or from different sound sources or tunes (‘different’ condition). Identification in each test was assessed by asking the subject to determine whether the source sounds or tunes for the members of each stimulus pair were the same or different, thereby avoiding the need for cross-modal labelling of particular sounds. The tests are schematised in Fig. 1 (stimulus details are in Supplementary Tables S2 and S3 on-line). Environmental sounds and melodies were all highly familiar to healthy older British individuals based on previous pilot work [4,5] (see also Supplementary Table S4 on-line). Stimuli were chosen such that ‘same’ and ‘different’ pairs did not differ in overall perceptual similarity: tunes were all presented as piano melodies controlled for musical attributes such as key, metre and tempo. The task therefore depended on semantic processing (identification of the source tune or sound) and could not rely simply on perceptual acoustic matching of paired sounds. Each test comprised 32 stimulus pairs comprising 16 ‘same’ trials and 16 ‘different’ trials. Further details about the experimental auditory tests are in Supplementary Material on-line.

### 2.3 Analysis

All experimental data were analysed in linear regression models using STATA12® with independent variables of group membership and test type: this design allowed assessment of interaction effects between tests and experimental groups, considering the combined patient cohort and the SD and TL groups separately versus the healthy control group. Age and years of musical training were included as nuisance covariates. Non-normally distributed neuropsychological data were analysed using Wilcoxon's rank sum test. Relations between experimental nonverbal auditory semantic tasks and standardised semantic and general cognitive measures (Tables S1 and S5) were assessed using Spearman's rho (r_s_) tests over the combined patient cohort. In addition, we performed a receiver-operating-characteristic (ROC) analysis to assess the potential diagnostic value of the novel nonverbal auditory semantic tests in discriminating patients from healthy participants. For all group comparisons, p < 0.05 was taken as the threshold criterion for statistical significance.

## 3 Results

### 3.1 Group data

Participant groups did not differ significantly in age, education, gender or musical background and patient groups did not differ in mean symptom duration (Table S1). The SD and TL groups displayed similar general neuropsychological profiles in line with their clinical syndromes, including significantly (p < 0.05) more severe impairment of verbal semantic functions in the SD group than the TL group. Participant performance profiles on the experimental auditory semantic tests are summarised in Table 1. On both experimental tasks, relative to healthy controls the combined patient cohort (environmental sounds: beta = − 4.5, p = 0.001; melodies: beta = − 6.8, p < 0.001) and both the SD group (environmental sounds: beta = − 3.8, p = 0.01; melodies: beta = − 6.2, p < 0.001) and the TL group (environmental sounds: beta = − 5.4, p = 0.004; melodies: beta = − 7.6, p < 0.001) showed comparable significant deficits. Allowing for the small case numbers involved, there were neither significant performance differences between the SD and TL groups nor between the SD group and the MAPT mutation subgroup (Table 1).

Scores on experimental auditory semantic tests were significantly positively correlated across the combined participant cohort (r_s_ = 0.55, p < 0.01), though not within the patient cohort (r_s_ = 0.43, p = 0.1); within the patient cohort, auditory nonverbal semantic performance was significantly positively correlated with performance on a standardised semantic memory measure (BPVS: r_s_ = 0.58, p = 0.02) but not with general cognitive capacity (MMSE; see Supplementary Table S5 on-line). Auditory apperceptive performance (indexed by PALPA-3) was intact in the TL group and did not correlate with environmental sound identification, suggesting that perceptual factors did not substantially confound the results.

**Fig. 2:**
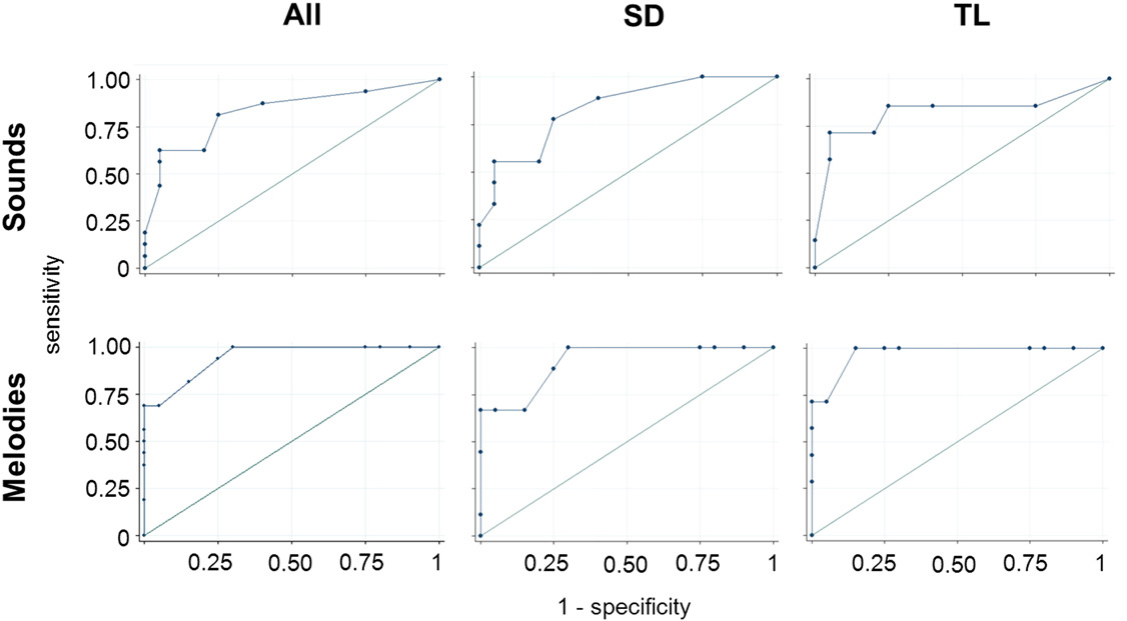
Results of ROC analysis for experimental tests assessing identification of environmental sounds (top panels) and melodies (lower panels) in the combined patient cohort (All) and in the semantic dementia (SD) and temporal lobe (TL) subgroups, as defined in the text. An ideal test (perfect discrimination of patients from healthy controls) would have a rectilinear profile; the diagonal line in each panel corresponds to chance level discrimination of patients from healthy controls.

Results of the ROC analysis are presented in Fig. 2. Considering the combined patient cohort, performance on both nonverbal auditory semantic tasks successfully discriminated patients from healthy participants (area-under-the-curve (AUC) for environmental sounds 0.83, p = 0.004; AUC for melodies 0.94, p = 0.008). Considering the syndromic groups separately, performance on the environmental sounds task successfully discriminated patients in both groups from healthy controls (for SD, AUC 0.84, p = 0.017; for TL, AUC 0.83, p = 0.01), while performance on the melodies task successfully discriminated patients in the SD group (AUC 0.93, p = 0.017) and showed a trend toward discrimination of patients in the TL group (AUC 0.97, p = 0.099) from healthy controls. A score < 29 on the environmental sounds task discriminated patients in both groups from healthy controls with > 75% sensitivity and specificity.

### 3.2 Individual profiles

Inspection of individual performance profiles (Supplementary Figures S2 and S3 on-line) revealed that patients generally performed better on the environmental sound identification test than the melody identification test. However, three patients (19% of cohort; one SD, two TL, one MAPT mutation) but only one (5%) of the healthy control participants performed substantially better for melodies. Two of these three patients had little or no musical training, arguing against an idiosyncratic effect of prior expertise. One patient exhibited musicophilia. All three patients showed substantial bilateral temporal lobe atrophy, with somewhat more marked involvement of the nondominant temporal lobe in one case (Figure S1).

## 4 Discussion

Here we have demonstrated that patients with clinically significant anterior temporal lobe atrophy have deficits of semantic processing in two key nonverbal auditory domains, identification of environmental sounds and melodies. The findings support earlier work [2,4,6], however our uniform, within-modality semantic classification paradigm allowed us to assess and compare auditory semantic domains more directly than has been possible previously. Whereas the SD group here showed more severe impairment of verbal semantic function than the TL group, nonverbal auditory semantic impairment was comparable in patients with anterior temporal lobe degeneration irrespective of whether they present with typical SD or with behavioural decline (and associated semantic impairment) underpinned by an alternative pathogenic process (such as MAPT mutations). Our findings corroborate previous neuropsychological evidence that the breakdown of semantic memory in SD is multi- or ultimately pan-modal [1,2,8,38] but further suggest that nonverbal sound may expose semantic deficits across syndromes. While conclusions must be tentative in the face of small case numbers and await direct anatomical substantiation, the present data are in keeping with disintegration of a common anterior temporal lobe semantic network in these syndromes [7,12,17].

While auditory semantic measures were correlated with general semantic competence in our patient cohort, individual patient performance dissociations (Figure S2) and the lack of correlation between environmental sound and melody identification performance (Table S5) suggest that semantic profiles for music versus other sounds may be separable in these syndromes. These data are consistent with the hypothesis that mechanisms subserving semantic knowledge across domains may reside in a distributed anterior temporal lobe network [1,38]. While preserved musical (melody) knowledge has not emerged as a group signature of SD here, patients who do exhibit such sparing represent important ‘test cases’ for understanding the cognitive architecture of semantic memory. From a clinical perspective, our data do not suggest that preserved melody knowledge is a necessary condition for development of musicophilia: two of the three patients who exhibited musicophilia here had impaired melody identification, suggesting that music may engage brain reward systems independently of accurate cognitive encoding [36]. A formal ROC analysis corroborated the clinical impression that auditory nonverbal impairment can assist diagnosis of patients with different clinical presentations underpinned by temporal lobe atrophy. These results should be regarded as preliminary: we suggest that the clinical value of assessing nonverbal auditory semantic function warrants further exploration as a potential adjunct to tests of verbal and visual semantic capacity that have been more widely applied in patients with dementia.

This study has several limitations that suggest additional directions for future work. Group sizes here were small, limiting power to detect effects: there is a need for larger cohort studies representing the wider spectrum of temporal lobe diseases (notably, Alzheimer's disease) and pooling data on genetic and other uncommon syndromes across specialist centres. The small TL group here encompassed substantial syndromic and pathological heterogeneity (in particular, patients with MAPT mutations versus sporadic bvFTD). The patients in this study might in effect be regarded as a series of single cases, each potentially informative in their own right. Analysis of larger cohorts might provide more fine grained auditory semantic signatures of particular disease groups, and group studies should be supplemented by detailed experimental investigation of individual patients. Such work (in particular, detailed study of individual dissociations) will be required to delineate critical relations between auditory and other semantic modalities and between auditory semantic and perceptual mechanisms. To define fully the brain substrates that mediate these cognitive operations is likely to require functional neuroimaging techniques, correlated with subsequent structural brain damage. Beyond elucidating the organisation of the human nonverbal semantic system, there is a clinical imperative to assess the diagnostic value of nonverbal auditory tests in relation to standard tests of semantic memory; and to determine how nonverbal deficits relate to daily life disability and symptoms that patients and their caregivers report. Longitudinal tracking of syndromes including the presymptomatic phase of genetically mediated diseases and ultimately, histopathological correlation will be essential to establish how deficits relate to one another and whether certain nonverbal semantic profiles might predict particular neurodegenerative pathologies or anticipate clinical evolution. Taking these caveats into account, our findings suggest that nonverbal auditory semantic impairment is a common feature of neurodegenerative syndromes with anterior temporal lobe atrophy, modulated by individual variation in the profile of auditory domains involved. The findings should motivate further study of this issue and more detailed exploration of its clinical significance.

## Conflict of interest

The authors have no conflicts of interest to declare.

## Figures and Tables

**Table 1:** Summary of group performance on experimental identification tasks.

Task	Healthy controls n = 20	All patients n = 16	SD n = 9	TL n = 7	MAPT n = 4
Environmental sounds (/32)	30.2 (2.0)	25.8 (4.3)*	26.4 (3.9)*	25.0 (5.0)*	26.5 (3.8)
Melodies (/32)	28.8 (1.9)	22.3 (3.7)*	22.8 (4.1)*	21.6 (3.4)*	22.5 (4.1)*

Group raw scores on experimental tests of sound and melody identification are shown (maximum score in parentheses after name of test); mean (standard deviation) values are presented. *significantly different from control group (no significant differences between groups or between conditions); SD, patient group with typical syndrome of semantic dementia (semantic variant of progressive aphasia); TL, other temporal lobe patient group; MAPT, subgroup of patients in the TL group with microtubule-associated protein tau mutations.
