# Supplementary material for: Identification of environmental sounds and melodies in syndromes of anterior temporal lobe degeneration
Source: J Neurol Sci. 2015 May 15;352(1-2):94–8. doi: 10.1016/j.jns.2015.03.007 (PMC4425361; doi:10.1016/j.jns.2015.03.007)
Supplement: Supplementary file 1 — Supplementary material. [file mmc1.docx]

**SUPPLEMENTARY MATERIAL**

**Identification of environmental sounds and melodies in syndromes of**

**anterior temporal lobe degeneration,** by HL Golden et al

**Details of experimental auditory semantic tests**

In each test, individual stimuli were relatively brief (see Tables S2 and S3) and paired stimuli on each trial were separated by a short inter-stimulus interval, in order to reduce working memory demands. Stimuli were equated for rms intensity value and overall duration across conditions.

Environmental sound and melody identification tests were presented sequentially; within each test, trials were presented in pseudorandomised order (each participant was presented the stimuli in the same, randomised order). Each individual stimulus was presented once in the ‘same’ and once in the ‘different’ condition. A notebook computer running the Cogent1.25® toolbox under MATLAB7.0® was used to administer sound stimuli as digital wavefiles and collect subject responses for off-line analysis. For each test, sound stimuli were presented at a comfortable listening level (at least 70 dB) in free field. Participants were first familiarised with the tests (using example stimuli that were not subsequently presented in the tests proper) to ensure they understood the task. For the environmental sounds test, the task on each trial was to decide whether the sounds in each pair were made by the same thing or by different things; for the melodies test, the task on each trial was to decide whether the tune excerpts in each pair were from the same song or different songs. During the tests participants received no feedback about their performance; each trial was presented only once, however no time limits on responses were imposed.

**Environmental sounds.** Environmental sounds representing a variety of familiar human, animal and inanimate sources were derived from a previously published battery (Goll et al., 2010a); sounds are listed in Supplementary Table S2 and examples are available from the authors. Sounds included were all highly familiar and readily identifiable to healthy older subjects based on previous pilot data in a separate healthy older cohort (Goll et al., 2010a). Sound pairs were constructed such that the acoustic similarity of the individual sounds comprising each pair did not differ systematically between the same and different conditions; on each trial, paired sounds were separated by a 1 second inter-stimulus interval.

**Melodies.** Tune pairs were created using Musescore® software; tunes are listed in Supplementary Table S3 and sound examples are available from the authors. Tunes included represented well-known classical and movie themes, carols, hymns and popular songs; all tunes included in the test were judged highly familiar to people of the participants’ background, based on pilot rating data in a separate group of healthy older British individuals (see Table S4). The procedure and some stimuli were adapted from previously published work (Omar et al., 2010). Stimuli were all composed in the same key (G major) using the same synthetic Musescore® piano timbre with pairs matched for tempo and metre. All melodies included in the test contained at least two, distinct but highly recognisable melodic motifs: for each melody pair, the tune excerpts included were chosen such that each excerpt was itself characteristic of the tune as a whole but the excerpts always differed melodically (see Figure 1). On each trial, paired melodies were separated by a 2 second inter-stimulus interval.

**Table S1. Demographic, clinical and general neuropsychological data for participant groups**

| **Characteristics** | **Healthy controls** | **SD** | **TL** |
| --- | --- | --- | --- |
| **General** |  |  |  |
| No. (m:f) | 20 (10:10) | 9 (4:5) | 7 (3:4) |
| Age (yrs) | 65.5(5.1) | 68.6(6.3) | 65.7(7.3) |
| Handedness (RH:LH)¶ | n/a | 8:1 | 6:1 |
| Education (yrs) | 16.2(2.6) | 14.1(2.4) | 15.3(3.1) |
| Musical training (yrs) | 1.8(2.6) | 2.4(3.8) | 3.7(4.6) |
| Music listening per week (hrs) | 7.8(8.6) | 3.7(4.9) | 4.9(5.0) |
| Symptom duration (yrs) | n/a | 5.1(2.6) | 5.4(3.5) |
| MMSE | n/a | 22.2(4.1) | 26.0(4.1) |
| **General neuropsychology** |  |  |  |
| ***Phonemic discrimination*** |  |  |  |
| PALPA-3 minimal word pairs (/36) | 35.6(0.9) | 34.2(1.9)* | 35.0(1.4) |
| ***Semantic memory*** |  |  |  |
| WASI Vocab (/80) | 71.9(4.5) | **28.2(18.7)**** | 53.6(16.0)* |
| WASI Similarities (/48) | 41.3(6.7) | **14.9(7.8)**** | 26.6(11.2)* |
| GNT (/30) | 25.9(3.1) | **0.8(2.3)**** | **5.4(6.1)*** |
| BPVS (/150) | 148.2(1.1) | **77.4(49.6)**** | **126.9(17.6)*** |
| Synonyms concrete | 24.1(0.8) | **14.7(6.4)*** | **19.9(4.3)*** |
| Synonyms abstract | 24.1(1.7) | **15.6(5.2)**** | 21.3(3.4)* |
| ***Episodic memory*** |  |  |  |
| RMT Faces (/50) | 45.2(3.9) | **34.1(7.3)*** | **31.6(8.4)*** |
| RMT Words (/50) | 48.2(2.6) | **29.9(7.5)**** | **34.1(4.0)*** |
| ***Executive*** |  |  |  |
| WASI Block design (/71) | 47.7(10.8) | 28.7(11.7)* | 25.6(15.1)* |
| WASI Matrices (/32) | 27.2(8.0) | 17.4(6.7)* | 16.3(6.8)* |
| D-KEFS Stroop^†^ colour (secs) | 30.6(5.3) | **48.9(23.6)*** | 39.7(22.9) |
| D-KEFS Stroop^†^ word (secs) | 21.4(4.0) | 28.9(9.2)** | 29.7(26.6) |
| D-KEFS Stroop^†^ interference (secs) | 53.7(9.7) | **93.8(32.4)*** | **89.6(45.7)*** |
| WMS-R digit span forward (/12) | 8.9(1.6) | 7.8(2.8) | 8.9(1.8) |
| WMS-R digit span reverse (/12) | 7.2(1.6) | 6.9(3.1) | 7.9(2.0) |
| ***Other functions*** |  |  |  |
| NART*^†^  (* /50) | 43.1(4.2) | 20.3(15.7)** | 34.1(12.7)* |
| GDA^††^ (/24) | 16.1(3.4) | 9.4(7.6)* | 12.4(3.6)* |
| VOSP Object Decision (/20) | 18.8(1.3) | 15.4(3.2)* | 15.1(5.2)* |
|  |  |  |  |

Values are mean(standard deviation, std) unless otherwise stated. Raw data are shown for neuropsychological tests (maximum scores in parentheses); results in bold indicate mean score <10^th^ percentile (using young adult norms for BPVS). Key: ¶assessed from clinical information; *significantly different from control group; **also significantly different between patient groups (p<0.05); ^†^7 SD patients completed this test; ^††^8 SD patients completed this test; BPVS, British Picture Vocabulary Scale (Dunn, Dunn & Whetton, 1982); D-KEFS, Delis Kaplan Executive System (Delis, Kaplan & Kramer, 1995); GDA, Graded Difficulty Arithmetic (Jackson & Warrington, 1986); GNT, Graded Naming Test (McKenna & Warrington, 1983); L>R, temporal lobe atrophy more marked on the left; LH, left-handed; MMSE, Mini-Mental State Examination score; n/a, not available; NART, National Adult Reading Test (Nelson, 1982); PALPA, Psycholinguistic Assessments of Language Processing in Aphasia, the written word minimal pairs were used (3) (Kay, Lesser & Coltheart,1992); R > L, temporal lobe atrophy more marked on the right; RH, right-handed; RMT, Recognition Memory Test (Warrington, 1984); SD, patient group with typical syndrome of semantic dementia (semantic variant of progressive aphasia); TL, other temporal lobe patient group (as defined in text), including 4 patients with MAPT mutations; VOSP, Visual Object and Spatial Perception Battery (Warrington & James, 1991); WASI, Wechsler Abbreviated Scale of Intelligence (Wechsler, 1999); WMS-R, Wechsler Memory Scale- Revised (Wechsler, 1987).

**Table S2. Environmental sound pairs in within-modality sound matching task**

| **Same sound source pairs** | | | | |
| --- | --- | --- | --- | --- |
| **Sound 1** | Duration (s) | **Sound 2** | Duration (s) | Diff (s) |
| cat howl | 5 | cat meow | 5 | 0 |
| horse neigh | 5 | horse gallop | 5 | 0 |
| female singing | 3 | female cough | 6 | 3 |
| male throat clear | 6 | male snore | 4 | 2 |
| cockerel call | 3 | cockerel clucking | 6 | 3 |
| male breathing | 4 | male yawn | 5 | 1 |
| water dripping | 7 | tap running | 9 | 2 |
| train horn | 4 | train engine | 6 | 2 |
| owl hooting | 6 | bird wings flapping | 7 | 1 |
| male whistle | 3 | male sneeze | 3 | 0 |
| telephone ring | 7 | telephone dial | 6 | 1 |
| door closing | 2 | door opening | 4 | 2 |
| coins jangling | 3 | coin dropping | 4 | 1 |
| car horn | 1 | car engine | 5 | 4 |
| male laughter | 4 | male sigh | 2 | 2 |
| dog pant | 8 | dog bark | 4 | 4 |
| *Mean(std) (secs)* |  |  |  | *1.8 (1.3)* |
|  |  |  |  |  |
| **Different sound source pairs** | | | | |
| **Sound 1** | Duration (s) | **Sound 2** | Duration (s) | Diff (s) |
| cat howl | 5 | male snore | 4 | 1 |
| horse neigh | 5 | female cough | 6 | 1 |
| female singing | 3 | horse gallop | 5 | 2 |
| male throat clear | 6 | cat meow | 5 | 1 |
| cockerel call | 3 | train engine | 6 | 3 |
| male breathing | 4 | tap running | 9 | 5 |
| water dripping | 7 | cockerel clucking | 6 | 1 |
| train horn | 4 | male yawn | 5 | 1 |
| owl hooting | 6 | male sneeze | 3 | 3 |
| male whistle | 3 | bird wings flapping | 7 | 4 |
| telephone ring | 7 | door opening | 4 | 3 |
| door closing | 2 | telephone dial | 6 | 4 |
| coins jangling | 3 | dog pant | 8 | 5 |
| car horn | 1 | male sigh | 2 | 1 |
| male laughter | 4 | car engine | 5 | 1 |
| dog bark | 4 | coin dropping | 4 | 0 |
| *Mean(std) (secs)* |  |  |  | *2.2 (1.6)* |

Mean pair duration differences not significantly different (p = 0.34) between same and different conditions. Dur, duration (seconds); Diff, duration difference between the sounds in the pair

**Table S3. Tune excerpt pairs in within-modality melody matching task**

| **Same source melody pairs** | | | | |
| --- | --- | --- | --- | --- |
| **Excerpt 1** | Dur (s) | **Excerpt 2** | Dur (s) | Diff (s) |
| The Four Seasons 1 | 7 | The Four Seasons 2 | 9 | 2 |
| Joy to the World 1 | 9 | Joy to the World 2 | 13 | 4 |
| Planets Jupiter 1 | 16 | Planets Jupiter | 16 | 0 |
| Brahms Lullaby 1 | 16 | Brahms Lullaby 2 | 16 | 0 |
| Star Wars Theme 1 | 10 | Star Wars Theme 2 | 10 | 0 |
| Auld Lang Syne 1 | 10 | Auld Lang Syne 2 | 10 | 0 |
| Land of Hope and Glory 1 | 9 | Land of Hope and Glory 2 | 9 | 0 |
| Hark the Herald Angels 1 | 9 | Hark the Herald Angels 2 | 18 | 9 |
| Que Sera Sera 1 | 8 | Que Sera Sera 2 | 14 | 6 |
| Silent Night 1 | 16 | Silent Night 2 | 21 | 5 |
| Frère Jacques 1 | 9 | Frère Jacques 2 | 9 | 0 |
| All Things Bright and Beautiful 1 | 17 | All Things Bright and Beautiful 2 | 17 | 0 |
| O Come All Ye Faithful 1 | 17 | O Come All Ye Faithful 2 | 17 | 0 |
| Away in a Manger 1 | 8 | Away in a Manger 2 | 8 | 0 |
| Amazing Grace 1 | 14 | Amazing Grace 2 | 14 | 0 |
| Jerusalem 1 | 16 | Jerusalem 2 | 17 | 1 |
| *Mean(std) (secs)* |  |  |  | *1.7 (2.7)* |
|  |  |  |  |  |
| **Different source melody pairs** | | | | |
| **Excerpt 1** | Dur (s) | **Excerpt 2** | Dur (s) | Diff (s) |
| The Four Seasons 1 | 7 | Joy to the World 2 | 13 | 6 |
| Joy to the World 1 | 9 | The Four Seasons 2 | 9 | 0 |
| Planets Jupiter 1 | 16 | Brahms Lullaby 2 | 16 | 0 |
| Brahms Lullaby 1 | 16 | Planets Jupiter 2 | 16 | 0 |
| Star Wars Theme 1 | 10 | Auld Lang Syne 2 | 10 | 0 |
| Auld Lang Syne 1 | 10 | Star Wars Theme 2 | 10 | 0 |
| Land of Hope and Glory 1 | 9 | Hark the Herald Angels 2 | 18 | 9 |
| Hark the Herald Angels 1 | 9 | Land of Hope and Glory 2 | 9 | 0 |
| Que Sera Sera 1 | 8 | Silent Night 2 (21s) | 21 | 13 |
| Silent Night 1 | 16 | Que Sera Sera 2 | 14 | 2 |
| Frère Jacques 1 | 9 | All Things Bright and Beautiful 2 | 17 | 8 |
| All Things Bright and Beautiful 1 | 17 | O Come All Ye Faithful 2 | 17 | 0 |
| O Come All Ye Faithful | 17 | Frère Jacques 2 | 9 | 8 |
| Away in a Manger 1 | 8 | Amazing Grace 2 | 14 | 6 |
| Amazing Grace 1 | 14 | Jerusalem 2 | 17 | 3 |
| Jerusalem 1 | 16 | Away in a Manger 2 | 8 | 8 |
| *Mean(std) (secs)* |  |  |  | *3.9 (4.2)* |

Mean pair duration differences not significantly different (p = 0.09) between same and different conditions. Dur, duration (seconds); Diff, duration difference between the sounds in the pair

**Table S4. Test melody familiarity ratings in healthy older controls**

| Tune | Mean (s.d.) |
| --- | --- |
|  |  |
| Amazing Grace | 5.0 (0) |
| Auld Lang Syne | 5.0 (0) |
| Away in a Manger | 5.0 (0) |
| Frère Jacques | 5.0 (0) |
| Hark the Herald Angels | 5.0 (0) |
| O Come All Ye Faithful | 5.0 (0) |
| Que Sera Sera | 5.0 (0) |
| Silent Night | 5.0 (0) |
| All Things Bright and Beautiful | 4.9 (0.3) |
| Land of Hope and Glory | 4.9 (0.3) |
| Brahms Lullaby | 4.8 (0.4) |
| Jerusalem | 4.8 (0.7) |
| Joy to the World | 4.8 (0.4) |
| Planets Jupiter | 4.8 (0.4) |
| Star Wars Theme | 4.4 (1.3) |
|  |  |

Based on pilot work in 9 healthy older British controls (age 50 – 72 years).

Rating scale graded 1(not at all familiar) to 5 (very familiar).

s.d., standard deviation

**Table S5. Correlations between experimental auditory semantic and standard tests**

|  | **Environmental sounds** | **Melodies** |
| --- | --- | --- |
| Melodies | 0.43 (0.1) | - |
| BPVS | **0.58 (0.02)** | 0.46 (0.07) |
| GNT* | 0.07 (0.79) | 0.43 (0.1) |
| Synonyms concrete | 0.45 (0.09) | **0.53 (0.04)** |
| Synonyms abstract | 0.37 (0.18) | 0.36 (0.19) |
| PALPA-3 | 0.30 (0.26) | **0.51 (0.04)** |
| MMSE | 0.21 (0.44) | 0.47 (0.07) |

Spearman’s rho (p-value) values of correlations between performance on experimental nonverbal auditory semantic tasks and standardised semantic and general cognitive indices in the combined patient cohort. Results in bold are significant at p<0.05 level; * likely affected by floor effects in semantic dementia (semantic variant primary progressive aphasia) group. BPVS, British Picture Vocabulary Scale; GNT, Graded Nmaing Test; MMSE, Mini-mental State Examination score; PALPA-3, Minimal Pairs Subtest from the Psycholinguistic Assessment of Language Processing in Aphasia.


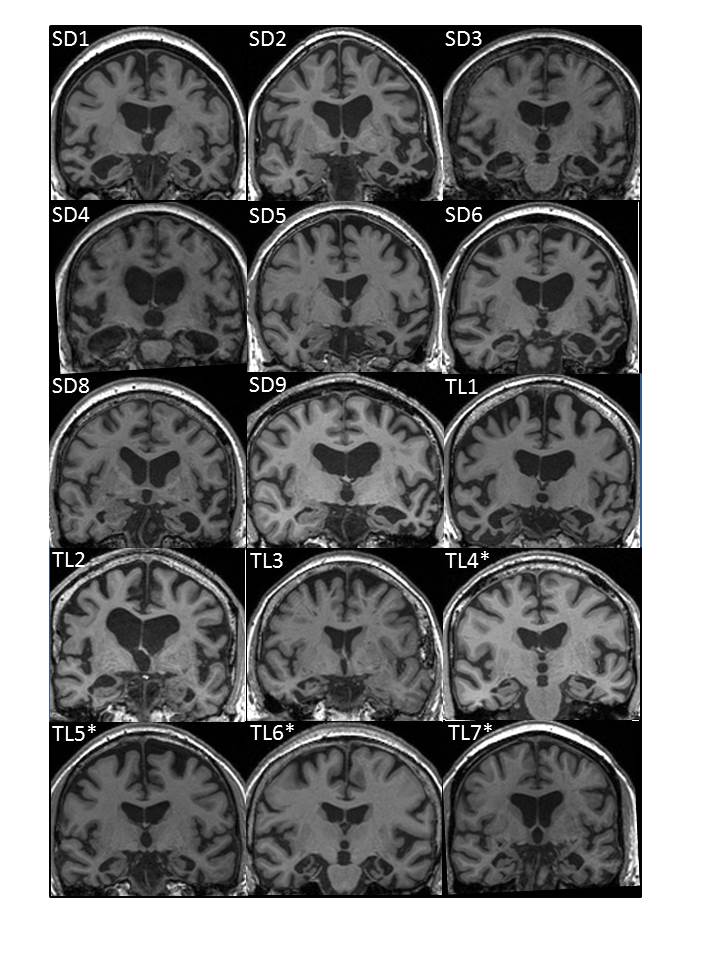


**Figure S1.** Representative coronal T1-weighted MRI brain sections for each patient. Images are displayed showing the left hemisphere on the right. SD, semantic dementia group; TL, other temporal lobe patient group (see text). Individual cases are coded as in Figure S3; *MAPT mutation. Cases SD4, TL2 and TL6 showed relative sparing of melody versus

environmental sound identification (see Figure S3).


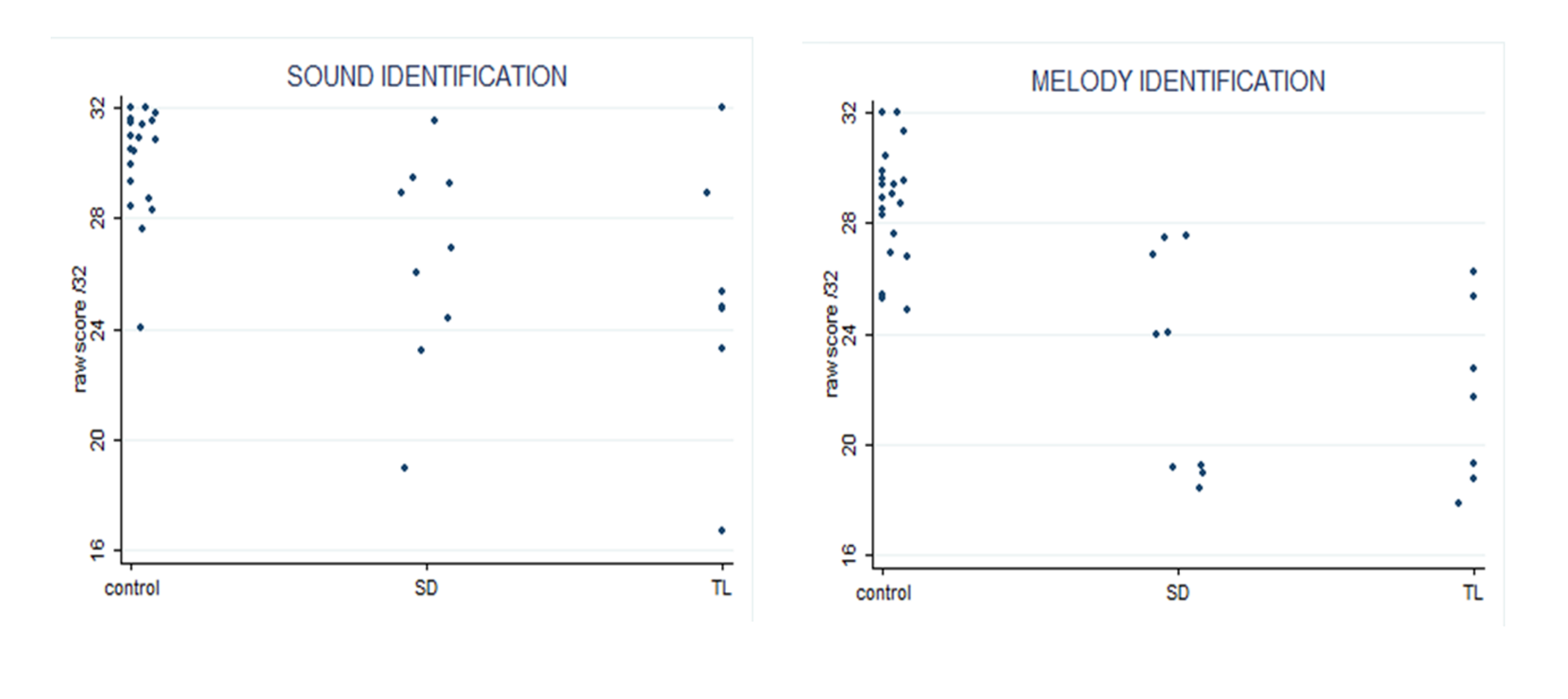


**Figure S2.** Individual raw scores for the environmental sound identification test (left) and the melody identification test (right). SD, patient group with typical syndrome of semantic dementia (semantic variant of progressive aphasia); TL, other temporal lobe patient group.

**
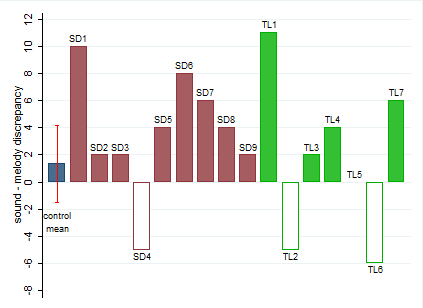
**

**Figure S3.** Discrepancies in raw scores between the environmental sound and melody identification tests for individual patients in the semantic dementia (SD, red) and temporal lobe (TL, green) patient groups; the mean score discrepancy for the healthy control group is also shown (blue). Data are referenced to a zero discrepancy line (corresponding to equal scores on both tests); data above the line indicate a higher score on the environmental sound identification test, data below the line indicate a higher score on the melody identification test. Error bar indicates standard deviation for the control group.
